# Envisaging challenges for the emerging medicinal Cannabis sector in Lesotho

**DOI:** 10.1186/s42238-024-00229-9

**Published:** 2024-05-16

**Authors:** Regina M. Thetsane

**Affiliations:** Department of Business Administration, National University of Lesotho, BTM – Building. Office Number BTM 201 P.0. Roma. 180, Maseru, Lesotho

**Keywords:** Medicinal Cannabis, Cannabis production, Cannabis industry

## Abstract

**Background:**

Cultivation of Cannabis and its use for medical purposes has existed for millennia on the African continent. The plant has also been widely consumed in the African continent since time immemorial. In particular, Lesotho has been largely growing Cannabis since approximately the 1550s and was illegally grown and unlawfully used for both medicinal and recreational purposes. It was only in 2017 when Lesotho started licensing Cannabis companies and regulating cultivation of Cannabis for medicinal purposes. However, the Lesotho Cannabis industry seems to have excluded the Small, Medium and Micro Enterprises (SMMEs) in the legalisation of Cannabis, the sector has the potential for small Cannabis enterprises in Lesotho.

**Objective:**

This study attempts to examine challenges facing the evolving Cannabis sector in Lesotho as envisaged by Cannabis company managers with the aim of being proactive while addressing such challenges.

**Methods:**

The qualitative descriptive method was employed using both primary and secondary data. For the selection of the three Cannabis managers exponential non-discriminative snowball sampling was adopted and interviews with the managers were recorded and transcribed verbatim. Thematic analysis was used to analyse the descriptive explanations of the Cannabis managers to determine the themes that were further consolidated into categories.

**Results:**

The implementation and compliance with the laws in the Lesotho medicinal Cannabis sector has proved very challenging, with long timeframes for finalising regulatory frameworks and not being applied objectively. The industry does not provide opportunities for Small, Medium and Micro Enterprises (SMMEs) to venture into the Cannabis business.

**Conclusion:**

In Lesotho, the Cannabis sector appears to be faced with many challenges emanating from the implementation and enforcement of Cannabis laws. The Lesotho Government should review its Cannabis laws and regulations with a view to benefiting SMMEs and legalising Cannabis production so as to serve both the domestic and international markets.

**Supplementary Information:**

The online version contains supplementary material available at 10.1186/s42238-024-00229-9.

## Introduction

The production of medical Cannabis in Lesotho was previously regulated under the Dangerous Medicines Act of 1973 (Government of Lesotho, 1978), which was superseded in 2008 by the Drugs of Abuse Act (Government of Lesotho 2008). This initiative allowed the Government to provide access to specific drugs for medical and scientific purposes (Chatwin 2017). Lesotho became the first in African country to change an existing legislation and decriminalise the cultivation of Cannabis for medical use (Prohibition Partners, 2019). The new law came into effect through the Drug of Abuse (Cannabis) Regulations Act of 2018 (Phakela 2018). Following the Kingdom of Lesotho, some African countries which legalised cannabis include, Uganda, Malawi, Swaziland, Zimbabwe and Morocco (Mkhize 2018). Cannabis production, with the highest levels of production globally taking place in Africa, has existed for millennia (Duvall 2019). For instance, ten thousand five hundred metric tons or approximately 25% of the global production of Cannabis herb reportedly took place in Africa in 2005 (United Nation’s Office on Drugs and Crime’s (UNODC, 2022)). While the highest rates have originated in West and Central Africa (13%) and in Southern Africa (8.5% (UNODC, 2022)), such, countries as, Ghana, Lesotho, South Africa and eSwatini have largely been growing Cannabis for ages (Prohibition Partners, 2019). Further, despite its use for recreational and medicinal purposes, the greater percentage of the Lesotho Cannabis has been illegal, the 70% of which has also been smuggled into the neighbouring South Africa (Deon 2021). Generally, Cannabis, which came to be known esoterically by local various constructs, be they, *matekoane, khomo ea fatše*, *likata* and *kakana*; elsewhere labelled marijuana, was found to be a more profitable crop that would enable Basotho to earn a better living than any other cash crops, such as, maize, sorghum and wheat. To date, many Basotho families have long been subsisting and collecting the extra income from selling Cannabis to both recreational and medicinal drug users to cover such additional costs as school fees for their children at different levels of schooling in Lesotho (Deon 2021).

Only in 2017 did Lesotho start licensing Cannabis companies and regulating the cultivation of Cannabis for medicinal purposes (Duvall 2019). Since then, the number of medicinal Cannabis businesses operating in Lesotho has grown rapidly following such a legislative change. In the same year, 2017, the first Lesotho company to be granted licences to produce Cannabis for medical and scientific purposes was the MG Health Limited Company. In the ensuing, the country has been experiencing an influx of foreign investment Cannabis companies. As Kabi (2018) stated that the Lesotho Ministry of Health (MoH) had granted thirty-three Cannabis licences across five licence categories: cultivation, processing, transport, retail and distribution in the same year.

Because of the influx of Cannabis companies, Lesotho has been projected to become a major source for medicinal Cannabis export into the markets of both Canada and the United Kingdom (UK), resulting in reducing poverty and unemployment in the country. This view is supported by Uwakonye (2020), who argues that in a resource-deficient country, for instance, Lesotho, the Cannabis industry may also upsurge the economy and become a solution to the high rate of youth unemployment (26.91%) (Aaron 2023). Considering the influx of medicinal Cannabis companies in Lesotho, the country’s economy, mainly driven by the construction industry, was projected to grow by 1.8 per cent in 2023, while the Cannabis industry was projected to contract (Central Bank of Lesotho, 2022), thereby rendering the future of this critically emerging sector rather oblique. Therefore, against this backdrop, this study set out to examine challenges facing the Cannabis sector in Lesotho.

## Literature review

### Cannabis

The most common Cannabis species grown around the world, including in Africa, and of course in Lesotho is *Cannabis sativa*, which can be traced to Asia (Government of Canada 2023). *Cannabis sativa* contains a complex mix of approximately 60 unique cannabinoids along with various chemical compounds (Western Cape Tourism, Trade and Investment Promotion Agency (Wessgro), (2021). The main active ingredient responsible for the production of Cannabis is delta-9-tetrahydrocannabinol (THC) (Wessgro), (2021). The National Institute on Drug Abuse (NIDA) (2019) also reported that the most commonly used parts of *Cannabis sativa* are dried leaves, flowers, stems and seeds and contain the mind-altering chemical THC and other similar compounds.

### Medical Cannabis product

The name Medical Cannabis relates to the therapeutic activity of herbal Cannabis and its constituents (Whiting et al. 2015). For Wessgro (2021), medicinal Cannabis is a legal, high-quality and standardised product made from crude Cannabis. Having started in China, Asia, then the Middle East, and Africa, the use of Cannabis for medicinal purposes spread to the rest of the world (Lafaye et al. 2017). As Bridgeman and Abazia (2017) pointed out, the Cannabis plant contains more than 100 different chemicals, known as cannabinoids, while Delta-9-Tetrahydrocannabinol (THC) and Cannabidiol (CBD) are the main relevant chemicals used in medicine. The authors further argued that THC makes an individual euphoric when smoking Cannabis or eating foodstuffs with cannabis contents. For the purpose of this research, medicinal Cannabis refers to plant-derived Cannabis products prescribed by medical practitioners for treating specific conditions as in epilepsy and pain (Bridgeman & Abazia 2017).

Cannabis can include high CBD and low THC products, although CBD products also appear as consumer goods. Medical Cannabis products are currently prepared as plant materials, oils, tinctures, edibles or capsules (Prohibition Partners, 2019). Medicinal Cannabis is administered with the intention of alleviating pains caused by diseases and illnesses, such as, Multiple Sclerosis (MS), depression, anxiety, Human Immunodeficiency Virus **(**HIV), nausea and vomiting, associated with chemotherapy, Posttraumatic Stress Disorder (PTSD), epilepsy or opioid addiction (National Academies of Sciences, Engineering, and Medicine (NASEM), 2017). However, medicinal Cannabis-related matters could be complicated in that, Cannabis is still an illegal drug and is legally constrained at the national and international levels in many countries (Mpela 2021). With some significant improvements in terms of legal imperatives by the industry, many challenges seem to lie ahead. Also noticeable worldwide is the increasing number of the Cannabis companies, making the industry highly competitive, something which has posed challenges for this particular industry. Doing business, coupled with observing the pertinent legal frameworks, using, distributing and growing Cannabis in such a legally multifaceted landscape, the Cannabis companies have encountered some difficulties (Matthew 2023).

### The challenges facing the Cannabis sector

#### The complex legal landscape

The medicinal Cannabis industry has been inhibited by regulatory restrictions for a long time. However, the legal market has emerged rapidly as many governments have started to legalise the production and use of medical Cannabis. For instance, many jurisdictions, especially in Europe, North America and South America, have liberalised control measures on Cannabis by decriminalising some instances of production, sales, possession and use. Some African countries, including Lesotho, have not been any exception to such a worldwide surge. Included in this industry are various activities, involving different professionals with direct, ancillary or tangential roles in the legal production, extraction, transport, sale and consumption or use of medical Cannabis, recreational Cannabis and any other related by-products (Duvall 2019). Further involve in cannabis industry are medical personnel, legal professionals, policy makers, dispensary owners and employees, cultivators and farmers, transport and handling personnel, and individuals as well as company manufacturing products as in oils and seeds for health and beauty products (Pacula and Smart 2017). However, with the industry being widely known for enforcing laws, some of which being clouded by ambiguous and contradictory regulations, Cannabis companies would end up operating with limited success (Bodwitch et al., 2019; Gianotti et al., 2017).

#### Cannabis cultivation methods

Medicinal Cannabis may grow either outdoors or indoors, thus differing from species to species as well as distinctive forms of produce, depending on different geographical situations. Outdoor cultivation is the traditional and original method of Cannabis cultivation that has been used for so long, mainly in African countries. Growing Cannabis outdoors exposes a crop to the elements, offering natural light and significantly reducing costs for growers. Nonetheless, the challenge is that Cannabis is exposed to harsh environmental conditions that may hinder an outdoor crop. For instance, prolonged heavy rains, insects and aggressive plants, such as, thistles, animals and extreme weather conditions are all potential crop killers. Improper soil and water resource management and pest control may also induce critical environmental hazards (Zheng et al. 2021). As a result, outdoor Cannabis growing could limit the growers’ control over environmental crossover from neighbouring fields.

In addition, medicinal Cannabis is mostly grown in green houses, mostly in developed countries. Joost (2019) reported that the intention is to mimic the elements of the outdoors that facilitate plant growth while maintaining full control over environmental parameters. The positive side of growing Cannabis in greenhouses is that the grower can detect how much carbon dioxide is in the air; how much moisture the plant needs; and even how well the soil conducts electricity (Bahji and Stephenson 2019). However, high upfront costs, including the building structures, equipment, water, electricity and other utilities, are the major downside of growing Cannabis indoors. Vaughan et al. (2021) argue that the light used indoors often reaches beyond greenhouses, causing some dissatisfaction on the part of the local communities and potentially disturbing ecosystem processes (Rich et al., 2020). Nonetheless, in 2019 and 2020, it was reported that growth in indoor Cannabis cultivation appeared to have overtaken growth in outdoor cultivation at the global level, with the overall net number of countries recording improved indoor cultivation being three times the net number of countries reporting a moribund outdoor cultivation (UNODC, 2022).

#### Competition

The common place increasing competition from Cannabis companies entering into the space is justifiably a major concern. The more countries legalise the medicinal Cannabis, the more companies enter into the Cannabis industry. For instance, on the African continent, Lesotho, Uganda, Malawi, Rwanda, Eswatini, Zimbabwe, Morocco and South Africa have already legalised the medicinal Cannabis. Such a series of legal frameworks in the Cannabis industry by the African countries may lead to a stiff competition among the Cannabis companies and across the African countries. A high competition may also threaten and push SMMEs Cannabis out of the emerging legal Cannabis industry (David et al. 2020).

#### Start-up costs

The Cannabis industry start-ups differ from traditional businesses with regard to the initial upfront costs. These are the expenses that Cannabis companies incur in the process of starting a new business venture. In many African countries, the expenses incurred include acquiring a licence fee for Cannabis production. The case in point is Lesotho where a licence fee for Cannabis production is approximately $350 000. In South Africa and Malawi, the production licence fee is approximately $1,465 and $10,000 respectively. In addition to the licence fee, setting up a medicinal Cannabis facility in South Africa, which cannot be afforded by many SMME Cannabis growers is estimated at $182,000 to $304 000. Adinoff and Reiman (2019) also argue that in the United States, economically disadvantaged individuals cannot participate in the legalised Cannabis market due to its high costs. In this view of this state of affairs, some researchers observe the participation of SMMEs Cannabis in the industry as crucial with potentially positive impact on the economy and citizens’ livelihoods in any given countries. This view is supported by Rusenga et al. (2022), who argue that legalising Cannabis production for medical purposes is extremely good. However, guaranteeing the involvement of ordinary citizens, SMMEs and local producers in the industry has to date proved challenging for many African countries.

#### The research capacity

In order to successfully inform health care decisions for a public policy, the research capacity of the Cannabis industry should be strengthened. Such policy-making decisions would require input from many stakeholders. These include clinical and public health Cannabis researchers; research methodologists; representatives from working groups who have developed research reporting guidelines; organisations engaged in standard development; representatives from scientific publications; and government agencies, with direct or indirect involvement in the research process (NASEM, 2017). In particular, institutions of higher learning should also come on board in advancing Cannabis research and offering courses geared towards the Cannabis industry.

Igiri et al. (2021) indicated that institutions of higher learning should offer courses, whose foci range from the business side of operations to growing and cultivating plants. Such courses could equip students with Cannabis consultancy, growing, technical extraction and dispensary operator skills. Establishing industry-focused universities for Cannabis research should be prioritised for the benefit of the country concerned. Besides, higher-learning institutions, particularly in Southern Africa, should be given funding for research, something has to date been a major barrier. Therefore, without adequate financial support, Cannabis research would hardly inform the health care and public health practice; nor would any initiatives for keeping pace with changes in Cannabis policy and patterns of Cannabis use make any headway. Supporting this view, Egbetokun et al. (2022) attribute low research output to poor funding and non-conducive and weak organisational climate in Africa. Some universities on the continent have few postgraduate programmes that further adversely affect their research output. Apart from lack of financial support for research and appropriate infrastructure, including laboratory facilities and equipment, the internet bandwidth required for collaboration with the global scholarly community, hence accessing more knowledge resources has been wanting (Igiri et al. 2021).

## Research methodology

Based on the qualitative design, the methodology used aimed to explore Cannabis managers’ experiences with the challenges in the emerging Cannabis industry in Lesotho. For secondary data collection, the study thus critically reviewed relevant documentary sources, while for primary data collection, semi-structured interviews each of which lasted from 10 to 15 min were conducted on a one-on-one basis in the Board room of each individual organisation. With the participants’ consent, the interviews were audio-recorded and transcribed verbatim for analysis. Ethical Approval was granted on the 28th August, 2020 by the Ministry of Health and Ethics Committee and the National University of Lesotho Institutional Review Board (IRB) REF: ID 94-2020.

### Respondents

The respondents for the study were the Cannabis companies’ managers, whose companies were registered, licenced and actively operated in Lesotho. There were eight registered and licenced Cannabis companies (Kabi 2018) which were all contacted for the interview. This information cannot be confirmed as the Chairperson of “Phekoane” (The Cannabis Association of Lesotho) could not provide the exact number of the registered Cannabis companies in Lesotho until the completion of the current study. The selection of the Cannabis managers adopted non-probability exponential non-discriminative snowball sampling, where the first manager, recruited to the sample group, provided multiple referrals, each of which was explored until the primary data from the adequate samples were collected (Mahin et al. 2017). This resulted in three Cannabis companies’ managers interviewed while five declined.

### Sampling

While it would be ideal to contact the entire target population for data collection, constrained resources, including time and access to certain participants became a factor. As such, sampling, particularly snowball sampling, was selected for this study. Snowball sampling is a purposeful method of data collection applied when samples with the target characteristics are not easily accessible (Vaughan et al. 2017). This technique was thus adopted for selecting the participant Cannabis managers from whom potentially rich data could be gathered for the study. The first Cannabis company manager contacted, provided multiple Cannabis managers who were followed up. Also, new referrals who accepted to participate pointed to more Cannabis managers, some of whose contact details were unavailable though. In the ensuing, the researcher arranged for the interview meetings with the readily available participants.

## Data processing and analysis

Premised on the qualitative approach, the interviews were transcribed verbatim and analysed. In the process, a thematic analysis was chosen as the analytical instrument for preserving the descriptions and explanations of the Cannabis managers rather than analysing the actual content or raw data for in-depth meanings (Vaismoradi et al. 2013). The first step was to review transcripts comprehensively and ascribe them into themes. This was followed by the process of abstraction and coding to label meaningful units, with the codes being arranged according to categories throughout the analysis. The analysed report was discussed with the managers for confirmation and providing feedback.

## The results

### Company managers’ demographic profiles

The data collection for this study drew on the participant managers, with the following demographic profiles: the managers’ gender, ages, current management positions, the highest qualifications and work experience in the management position. The summarised demographic profiles of each interviewed manager are presented in Table 1.1.

**Table 1.1:** Demographic characteristics of the target managers (*N* = 3)

		Number	%
Gender	Male	2	66.67
	Female	1	33.33
		**3**	**100**
Age	(18–24)	0	0
	(25–34)	1	33.33
	(35–44)	1	33.33
	(45–54)	0	0
	(55–64)	1	33.34
		**3**	**100**
Current Management Position	Operations Manager	0	0
	Cultivation Manager	3	100
	Manufacturing Manager	0	0
	Extraction Manager	0	0
	Other	0	0
		**3**	**100**
Highest Qualification	High School	0	0
	Diploma	0	0
	Associate Degree	0	0
	Bachelor’s Degree	1	33.33
	Master’s	2	66.67
	Doctorate (PhD)	0	0
		**3**	**100**
Area of Specialisation	Agriculture	2	66.67
	Agriculture and Entrepreneurship	1	33.33
	Plant Science & Horticulture	0	0
	Cannabis Studies	0	0
	Pharmacology and Pharmacy	0	0
	Biology	0	0
	Business Administration	0	0
		**3**	**100**
Year of Experience in the Position	1–5 years	3	100
	6–10	0	0
	11–15	0	0
	16–20	0	0
	21+	0	0
		**3**	**100**

Author compilation (2023)

As shown in Table 1.1, the age of the three managers interviewed ranged from 25 to 64. All the participants were managers in the Cultivation Department. This might be because the three companies in which the managers agreed to be interviewed were at their initial stage of Cannabis production (cultivation). Regarding the level of education, the two managers each had a master’s degree, while one had a first degree. Further noted is that the closest master’s degree to Cannabis cultivation held by one of the respondents is Master’s in Agriculture. All the target managers had three years of experience in cultivation management.

### Challenges for the emerging medicinal Cannabis sector

Challenges for the emerging medicinal Cannabis sector were reported as having varied categories. These include the ‘Legislation framework, interpretation and implementation’, ‘non-conformity to medical Cannabis regulations’, ‘Lack of accredited laboratories’ ‘Irrelevant skills’, ‘Accessibility of the Cannabis international markets’ and ‘Cannabis licence fees’. The responses, composed of verbatim statements made by the focal Cannabis cultivation managers are discussed below:

#### The legislation framework, interpretation and implementation

The respondents point to an array of limitations on the Lesotho Cannabis industry legislation, including its enforcement and implementation.

***For example, as noted by Respondent 1***:

*The legislation governing the Cannabis industry in Lesotho is unclear, difficult to comprehend, and it is not applied fairly.*

***Respondent 3 also commented***:

*I am wondering if these people who are in charge of the legal aspect of the Cannabis were ever exposed to some training on Cannabis laws and regulations under the auspices of the Ministry of Health. These people know nothing about Cannabis laws in the country.*

This theme highlights the importance of careful interpretation and implementation of Cannabis laws and regulations in Lesotho.

#### Non-conformances to medical Cannabis regulations

The theme basically relates to non-conformity to medical rules and regulations. Crucial here is the first theme, legislation interpretation and implementation. The respondents observed the staff in the Ministry of Health as not complying with the rules and regulations of the industry. Such non-compliance may negatively impact on the industry, thus rendering the country at risk of being blacklisted by the International Narcotics Control Board (INCB).

***In amplification of the above***, **Respondent** 2 ***had this to say***:

*The staff in the Ministry do not have any interest in the industry. This is demonstrated by a lack of conformity to the industry regulations. As it stands, Lesotho is at risk of being blacklisted in the International Narcotics Control Board (INCB) because the Ministry have to date not being able to provide required annual reports.*

***Responded 3 remarked thus***:

*Those people in the Ministry are always sitting in offices; they do not go out to inspect and collect figures of the companies harvest. Nor do they know how much Cannabis Lesotho is producing. I won’t be surprised if people are still smuggling our Cannabis.*

This theme suggests that the industry should adapt to a fast-changing, complex Cannabis legal landscape. Strict regulatory measures are required to protect both the domestic and international Cannabis markets and support the transition of small-scale illegal cultivators to the legal regime.

### Lack of accredited laboratories

With the Cannabis industry finally in the legal realm in Lesotho, Cannabis testing has emerged as key to the emerging Cannabis sector. Cannabis testing enables growers, distributors and sellers to observe the Lesotho Cannabis regulations, while offering the highest quality, consistent and easy-to-dose products that are safely consumable to the customers. To this end, since Lesotho does not currently have its own testing laboratories, having all the companies take the samples to South Africa for Cannabis testing has been considered very expensive by the respondents.

*Respondent 1 commented thus*:

*There are no accredited laboratories for testing the levels of* Tetrahydrocannabinol *(THC) and* Cannabidiol *(CBD) in our Cannabis products. Thus, the samples are taken to foreign countries for tests, something which turns out to be expensive for our companies.*

***Respondent 3 had this to say***:

*Some small Cannabis companies do not test their products at all because they cannot afford to take their Cannabis to South Africa. The laboratory that was located in the Letšeng Diamond Mining Company is not working. How could we have good cannabis produce while we do not have any testing laboratories?*

### Lack of relevant skills

The Cannabis industry in Lesotho is under the auspices of the Ministry of Health. The respondents observed that the employees attached to the Department of Cannabis in the Ministry do not have requisite skills and competencies.

***In support of their views respondent 1 had this to say***:

*The Lesotho Health Ministry staff are very incompetent and do not have any interest and skills to run the sector. Neither are they willing to learn about the Cannabis industry, yet they are required to assist us with regulations related to licences, production, manufacturing, distribution and retailing of Cannabis. There is also a limited number of manufacturing pharmacists with comprehensive skills in the Cannabis industry, coupled with lack of compliance with Good Manufacturing Practices (GMP) and (GACP) Good Agricultural and Collection Practices and Good Manufacturing.*

The other two respondents commented on the importance of training employees on Cannabis-related courses.

***Respondent 1 stated as follows***:

*Our higher-learning institutions should introduce courses on Cannabis plants, including Cannabis short courses, targeting Cannabis employees who lack cannabis knowledge. Programme review should be done so that the country can provide Cannabis programmes needed by the country.*

***Respondent 2 remarked***:

*Cannabis companies should also offer their employees scholarships to further their studies, particularly in areas related to Cannabis production, Cannabis products and cultivation security. It could even be mandatory for all the Cannabis companies to provide hands-on experience for their employees in documenting the whole process, including growing the plant.*

This theme suggests the importance of training employees to acquire requisite and relevant skills and competencies for the newly emerging Cannabis industry.

### The international Cannabis markets

Currently, Lesotho has only one company producing and manufacturing Cannabis. It is the only one company which export the medicinal Cannabis flower as an active pharmaceutical ingredient (API) to the EU market. This means that the majority of the Lesotho Cannabis companies have, to date, not complied with the GMP requirements. As such, they cannot export the Lesotho Cannabis products to the international market.

***Respondent 1 had this to say***:

*Without being granted the GMP (Good manufacturing practices and GACP (Good Agricultural and Collection Practices), Lesotho cannot sell anywhere in the world, nor can the country export to and access the international Cannabis markets.*

This theme advocates for the improvement of the production of Cannabis in Lesotho in order to meet international standards for being granted the GMP and GACP. This theme relates to adhering and conforming to the rules and regulations governing the Cannabis industry.

### Cannabis licence fees

The theme relating to the Cannabis licence fee is very important, as it affects the establishment of Cannabis companies in Lesotho. The Basotho people cannot venture into not afford operating in the Cannabis industry mainly because of the initial costly licencing. This indicates that although the 2008 legislation made it possible to grow Cannabis for medicinal purposes in Lesotho, growing it even for recreational purposes, without the licence from the Lesotho Ministry of Health still remains illegal in the country.

**As*Respondent 3 indicated***:

*It is not easy for the Basotho to establish their own Cannabis companies because the Cannabis licence fee is very expensive in Lesotho. Only the elite and multinationals have benefited from the Cannabis legislation that was heralded as something that would spread the economic gains amongst the Basotho.*

This theme could be seen as the industry denying the local Basotho an opportunity to venture into the Cannabis business. Rather, the industry could be considered to be reserved for only few individuals with the potential or capacity to raise the required capital. The majority of the Basotho who have been cultivating Cannabis since time immemorial have apparently been excluded if not constrained by such Cannabis regulatory frameworks in Lesotho. Of the most favoured by the current or emerging Cannabis regulatory frameworks seem to be the large businesses over the smallholder farmers in the country.

## Discussion

Despite mixed experiences and limitations the Cannabis managers shared several challenges faced by the emerging Cannabis industry in Lesotho.

### Industry legislation and compliance

The Cannabis industry in Lesotho is still at the rudimentary stage. Therefore, it is instructive to highlight, as reported by the respondents, that the legislation governing the industry is not yet refined nor fairly implemented. As observed globally, the scenario of Lesotho regarding the implementation of and compliance with the laws in the medicinal Cannabis sector has posed some challenges. Long timeframes for finalising regulatory frameworks have also met with problems of objective application (Rychert et al. 2021). However, due to the nature of this Cannabis products, the legality and regulation of the Cannabis industry is crucial. Regulatory bodies around the world have been instituting policies and guidelines to help to ensure safe and effective Cannabis production. Therefore, Lesotho is no exception as a member of the International Narcotics Control Board (INCB). The INCB responsibility is to administer a system of estimates for narcotic drugs and a voluntary assessment system for psychotropic substances, and to monitor licit activities involving drugs through a statistical returns system, with a view to assisting governments towards achieving, inter alia, a balance between supply and demand (INCB, 2011). Consequently, Lesotho is expected to enforce and adhere to the Cannabis laws, rules and regulations and duly submit regular reports regarding the Cannabis status for the INCB’s consideration.

The growing demand for medicinal Cannabis in Lesotho has created a booming industry that requires relevant and requisite skills. Taking the point further De Gobbi et al. (2022), highlights the Lesotho’s opportunity for the booming Cannabis sector for which requisite skills and knowledge could be inculcated among the Basotho for their vigorous involvement in the industry. Such skills could benefit the Basotho, including Cannabis cultivators and the Lesotho legal framework personnel. However, Letete (2023) reported that the Lesotho Cannabis industry has not lived up to the Basotho expectations. Small-scale growers are excluded from the emerging Cannabis industry, expatiating their illegal subsistence farming activities even more risky. As noted earlier, participation in the Cannabis sector, especially its licence fees, has become unaffordable for many Basotho interested in dealing in the Cannabis industry. Kabi (2018) attested that of the 33 people who had acquired licences to grow medical marijuana, only 13 had then paid M540, 000 ($28.18) for their operators’ licences. Concurring, Bloomer (2019) saw the current start-up costs for Cannabis production as being extremely high, thus excluding SMME participation in the industry in some African countries, including Lesotho and Malawi. This might be because, in particular, the Lesotho government has not done much to improve the possibilities of those who are already farming Cannabis inside the Lesotho legal framework.

### Accredited testing laboratories

The Cannabis industry is a fast-paced industry, with the constantly changing job market, which requires different infrastructure and skill demands. One of the main requirements for effective Cannabis production are readily available and accredited test laboratories. With such test laboratories, determining potency of Cannabis products could be easily confirmed to avoid any harmful contaminants and ensure regulatory limits. Josh (2021) confirmed that Cannabis is predominantly tested for accurate labels and safety before consumption. On the other hand, Cannabis operators use testing results to improve their processes and to monitor the product quality versus state-specific regulatory requirements that could cause their products to pass or fail. It is, therefore, mandatory for countries, including Lesotho to provide Cannabis-testing infrastructure and ensure consumption of accurately labelled and uncontaminated products.

### The industry-oriented skills

The Cannabis industry emanates from science-based to business-based careers, and the skills needed for each Cannabis job vary greatly (Fong, 2020). However, this fast-paced industry, with its constantly changing job market and varied skill demands, needs some of the most in-demand Cannabis skills as in basic Cannabis knowledge, reading and writing, basic computer and general skills. Jobs beyond an extractor require a bachelor’s degree in such areas as business, engineering, biology, chemistry, agriculture and horticulture, while scientific careers will require a master’s and doctorates. Careers related to law and policy, criminal justice and pharmacy are interconnected with Cannabis. Health professionals would especially need more education to ensure that they understand the medical benefits (Dattani 2018). On this basis, local institutions should introduce programmes that are geared towards the Cannabis industry skills. Cannabis SMMEs who may want to enter the Cannabis industry should also be provided with relevant training for participating in the industry.

## Limitations of the study

As in any other study, some limitations have been noted in this particular study. This was all the more so, considering that the Cannabis industry was primarily in its infancy stage during the fieldwork for this study in the country. While the coming into being of the industry somewhat spurred this research study, at the time, some of the potentially relevant and information-rich participants could not come forward for providing data as expected. Neither could the precise number of the registered Cannabis companies be determined in the Kingdom of Lesotho at the time. Only a few companies appeared to have registered albeit not being fully operational. On the face of it, and of course, without necessarily undervaluing the phenomenon reported in this study, the snowball sampling technique, coupled with purposive sampling, were judiciously adopted for data collection. As such, from the purposively sampled target Cannabis managers, the study has been able to present, analyse and interpret the data which, as revealed, could shed light on the phenomena under study. Of course, with such a small sample, largely typical of the qualitative research adopted here, the findings may not be generalised to other contexts. Nevertheless, it is hoped that from the findings, conclusive remarks on the phenomenon of Cannabis production and its related legislative imperatives in the Kingdom of Lesotho could be made. From such conclusions, as noted below, future studies could carry over for further interrogating the Cannabis industry in Lesotho and possibly elsewhere.

## Conclusions and recommendations

Considering the above findings, conclusions can be drawn based on the aim of the study. Conducting interviews with the Cannabis company managers, the study set out to identify challenges facing the Cannabis sector in Lesotho. The paper has found majority of the Lesotho Cannabis challenges as emanating from the legal framework governing the Cannabis sector in Lesotho. Inconsistencies in implementing and complying with the Cannabis laws and regulations by the Government Ministry of Health employees have also been noted. Issuing people with medicinal Cannabis licences has been done without any follow up on their use on the ground. At the time of study, Lesotho had thirty-three Cannabis companies, eight (8) of which were legally registered, and only one (1) had been granted Good Manufacturing Practices (GMP) certification. The GMP is a certification granted to producers who have stuck to the guidelines and regulations for dealing in Cannabis products according to GMP (Novak and Iguera 2019). This could indicate that Cannabis companies in Lesotho do not fully comply with the laws and regulations of the sector, resulting in only one Cannabis company being able to export Cannabis to EU markets. However, the export of medicinal Cannabis is necessary because not all countries have legalised medicinal Cannabis. However, the countries with legalised medicinal Cannabis apparently have limited domestic production capacity. For Lesotho to benefit from the Cannabis industry, strict compliance with the Cannabis laws is crucial.

The higher-learning institutions in Lesotho have the potential to support Cannabis career pathways by introducing and providing Cannabis-related programmes, thus assisting in producing a skilled and knowledgeable Cannabis workforce. However, also critical for the ultimate transfer of medicinal Cannabis skills and standards to the locals is the commercial knowledge of the international investors in the country. As such, the government should take measures to strengthen linkages with the medical Cannabis industry and promote economic activities so as to create low-skill jobs for small-scale farmers. With such newly created low-skill jobs, the locals should be retrained to work in the medical Cannabis industry, with the foreign workers being recruited largely or only for rare and/or special skills.

Cannabis production is increasingly becoming critical to Lesotho and to the international community. Therefore, the legalisation of the medicinal Cannabis sector should be reviewed so as to involve relevant stakeholders in Lesotho. In particular, the Lesotho Government should urgently spearhead such a review of the Cannabis laws and regulations for benefit of the entire Basotho nation, including the SMMEs, thus turning the extant illegal growers into legal growers who could also respond to the domestic marketing demands. As noted earlier, the Lesotho Government could thus formulate clear regulatory infrastructure to ensure responsive and sustainable Cannabis industry. Finally, liaising with higher-learning institutions, particularly in the country, the Government could develop requisite talents among the interested citizens, who would, in turn, be vigorously involved in the industry in Lesotho. Such strides could consolidate the Lesotho’s position as the leading trendsetter against which other African countries may benchmark.

### Electronic supplementary material

Below is the link to the electronic supplementary material.

Supplementary Material 1

## Abbreviations

API: An Active Pharmaceutical Ingredient
CBD: Cannabidiol
GMP: Good Manufacturing Practices
HPS: High Pressure Sodium Light
INCB: International Narcotics Control Board
LED: Light-emitting Diode
MoH: Ministry of Health
NASEM: National Academies of Sciences, Engineering, and Medicine
NIDA: National Institute on Drug Abuse
PhD: Doctor of Philosophy
GACP: Good Agricultural and Collection Practice
SMMEs: Small, Medium and Microenterprises
THC: Tetrahydrocannabinol
UNODC: United Nations Office on Drugs and Crime
USA: United States of America
