# Supplementary material for: Envisaging challenges for the emerging medicinal Cannabis sector in Lesotho
Source: J Cannabis Res. 2024 May 16;6:23. doi: 10.1186/s42238-024-00229-9 (PMC11097424; doi:10.1186/s42238-024-00229-9)
Supplement: Supplementary file 1 — Supplementary Material 1 [file 42238_2024_229_MOESM1_ESM.pdf]

The National University of Lesotho

Telephone: +26622340601
Fax: +266 22340000
http://www.nul.ls

P.O. Roma 180
Lesotho 29/02/2024

Faculty of Humanities

TO WHOM IT MAY CONCERN

This letter serves to confirm that I have edited the manuscript with the title***:*** *Envisaging challenges for the emerging Medicinal Cannabis sector in Lesotho*.

I am an English language practitioner, a professional editor and proofreader with academic literacy and editing skills as well as scholarly peer-reviewing experience, based at the above-mentioned institution. Should there be any queries, please feel free to contact me for clarity.

I look forward to your usual co-operation, thus thanking you in advance.

Yours faithfully

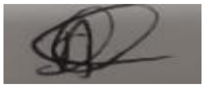

Mokhoele Aaron Hala-hala (PhD)
Senior Lecturer and Co-ordinator of the Communication & Study Skills Unit
The Department of English
The National University of Lesotho
P. O. Roma 180
ma.hala-hala@nul.ls
mokhoeleahalahala@gmail.com
Cell +266 58403924/63832503
